# The Long and Winding Road (Part 2)

**DOI:** 10.5812/traumamon.9497

**Published:** 2013-01-15

**Authors:** Shahram Nazerani

**Affiliations:** 1 Department of Surgery, Firouzgar Medical Center, Tehran Medical Sciences University, Tehran, IR Iran

**Keywords:** Education, Medical, Student

After obtaining a Medical Degree following years of study and the subsequent long and exhausting specialty education program, the tired and now not-so-young doctor has to ship-off to rural areas and practice in a small town in order to gather “Points” compulsory for a Permit to practice medicine in his hometown or major cities. Years later, after obtaining the Permit he returns to his hometown and buys a new, albeit small apartment with the meager money saved from the sojourn in rural areas plus of course, a major loan from his father!

One day, after coming home exhausted from an especially tiring shift at the hospital he flops down on the sofa. He feels a cold draft and starts to shiver; he looks around the apartment and wonders why the newly built flat has no modern amenities such as Energy Efficient Construction or new technologies for climate control? He finds that lack of insulation is the cause of cold drafts from every window and door; dust gathers in the apartment and the heating system cannot cope with temperature demand and energy loss due to faulty engineering. The doctor wonders why the engineer who built the apartment had no knowledge of the new technologies such as the “Energy Efficient House” which has nearly no energy leak or the Geothermal-Solar Energy House to generate heat especially in a country with 320 days of sunshine! (1)

Why is it that we as physicians must strive to learn, stay up-to-date, know the best treatment modalities, use the latest equipment, meet the demands of our patients, earn Continuing Educational Credits to keep our offices open, be tried by professional peers who rule by the latest standards of medicine for our mistakes and have to pay dearly if litigated, whilst engineers do not? Shouldn’t Engineers who do not have to work for years in the rural areas in order to get a Permit to work in major cities or their hometown, who knowingly or unknowingly, neglect modern day technologies in their construction be responsible for their actions also? Shouldn’t they have to uphold their commitment to their community and society? Old and unsafe construction and faulty insulation standards are responsible for the mind-blowing costs of electricity and gas to warm or cool these sieves they call flats while wasting the nonrenewable and precious energy resources namely fossil fuels in addition to adding more pollution to the already polluted cities. Aside from this issue the quality of construction is also important. God forbid, if a strong earthquake hits, who will be held responsible for the collapse and massive casualties incurred by these “cardboard box” houses they build? (2, 3)

We have a very strong ruling Medical Judiciary Council to control our standards of medical care; they judge us according to the latest standards of health management. I think engineers and architects should strictly abide by contemporary quality control measures or likewise face judicial penalties. Although much has been done by the municipality with regard to engineering, much more needs to be done
